# Role and Mechanism of Microglia in White Matter Injury Recovery in Ischemic Stroke

**DOI:** 10.1002/iid3.70226

**Published:** 2025-08-27

**Authors:** Yi‐Sha Guo, Yunlin Shang, Jiajia Yao, Xia Bi

**Affiliations:** ^1^ Department of Physical Therapy Affiliated Yangzhi Rehabilitation Hospital of Tongji University, Shanghai Yangzhi Rehabilitation Hospital Shanghai China; ^2^ Department of Rehabilitation Medicine Shanghai University of Medicine & Health Sciences Affiliated Zhoupu Hospital Shanghai China

**Keywords:** ischemic stroke, microglia, myelin, oligdendrocytes, white matter

## Abstract

**Background:**

Ischemic stroke frequently leads to white matter injury (WMI), significantly impairing neurological function and recovery. Microglia, the central nervous system's resident immune cells, play a dual role in poststroke pathology and repair. Their diverse activation states and interactions with other glial cells influence demyelination, remyelination, and overall WMI outcomes. To systematically review and synthesize the current evidence regarding the temporal and functional dynamics of microglia in ischemic stroke, with a focus on their roles in white matter damage and recovery.

**Methods:**

A comprehensive literature search was conducted using PubMed, Web of Science, and Scopus databases (through December 2024) with keywords including “ischemic stroke,” “white matter injury,” “microglia,” “myelin,” and “oligodendrocytes.” Studies involving mechanistic insights into microglial polarization, myelin repair, and associated molecular pathways were prioritized. Both preclinical and clinical studies were reviewed.

**Results:**

Microglia exhibit distinct activation profiles in acute and chronic phases poststroke. Pro‐inflammatory microglia exacerbate WMI via cytokine secretion and oligodendrocyte toxicity, while immune‐regulatory microglia promote remyelination through trophic support and debris clearance. Key regulatory pathways include TREM2, CX3CR1, and purinergic signaling. Microglial phagocytosis, cytokine production, and interactions with astrocytes critically modulate remyelination. Therapeutic modulation of microglial phenotype (e.g., fingolimod, HDAC inhibitors) shows promise in enhancing white matter repair.

**Conclusion:**

Microglia exert time‐ and region‐specific effects on WMI after ischemic stroke. A nuanced understanding of their dynamic phenotypes and interactions with other glial elements is essential for developing targeted therapies. Future research should integrate single‐cell technologies, human validation, and sex‐specific analyses to refine microglia‐based interventions.

## Graphical abstract

Many studies have shown that stroke can cause microglia activation, and activated microglia differentiate into different types according to different time and region, and different types of microglia play an important role in the process of white matter recovery.

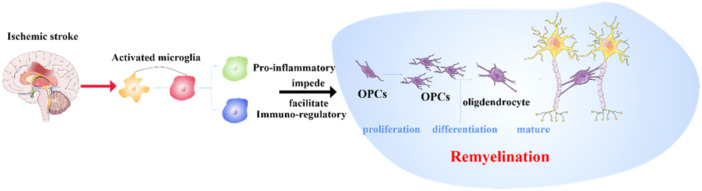

## 1 Introduction

Stroke is one of the leading causes of death and disability worldwide and imposes a significant health and economic burden worldwide, with an annual mortality rate of about 5.5 million and disability accounting for 50% of survivors [1]. Ischemic stroke, accounting for ~87% of all strokes, is not a homogeneous entity but rather a heterogeneous disease with distinct etiopathogenic subtypes, including atherothrombotic infarcts, cardioembolic strokes, lacunar infarcts, and infarcts of unusual or undetermined etiology [2, 3]. These subtypes differ critically in their underlying mechanisms, risk factor profiles, and clinical outcomes. For instance, lacunar infarcts, often linked to cerebral small vessel disease, are strongly associated with hypertension and present unique neuroimaging and prognostic characteristics, while cardioembolic strokes frequently involve atrial fibrillation and exhibit higher initial severity [4, 5]. Such heterogeneity underscores the necessity of subtype‐specific analyses in both preclinical and clinical research, as therapeutic strategies targeting neuroinflammation, neuroprotection, or white matter repair may vary depending on the ischemic insult's origin and pathophysiology [6, 7, 8].

The differences in microglial responses between atherothrombotic and lacunar infarcts can be attributed to various factors, including the duration of ischemia, the inflammatory milieu, and the integrity of the blood‐brain barrier. In atherothrombotic infarcts, which are typically larger and more severe, the duration of ischemia plays a significant role in the microglial response. The longer the ischemic duration, the more profound the microglial activation and subsequent neuroinflammation. This is supported by the finding that higher cardio‐ankle vascular index and higher uric acid levels, which are associated with prolonged ischemia, were independently associated with infarct expansion in lacunar infarction patients, as reported by Sato et al. [9].

On the other hand, lacunar infarcts, which result from the occlusion of small penetrating arteries, exhibit a different pattern of microglial response. In these infarcts, the inflammatory milieu and the integrity of the blood–brain barrier play more critical roles. The lower/normal low‐density lipoprotein (LDL)‐cholesterol levels were found to be associated with infarct expansion in lacunar infarction patients, suggesting a potential link between cholesterol metabolism and microglial activation in this context [9].

The inflammatory response in lacunar infarcts is also influenced by the blood–brain barrier integrity. Disruption of the blood–brain barrier can lead to increased infiltration of peripheral immune cells and subsequent exacerbation of neuroinflammation. In contrast, atherothrombotic infarcts are characterized by a more robust inflammatory response, involving both microglial activation and peripheral immune cell infiltration. This is consistent with the finding that higher LDL‐cholesterol levels, which are associated with a more severe inflammatory response, were independently associated with infarct expansion in giant lacunar infarction patients.

Overall, the microglial responses in atherothrombotic versus lacunar infarcts are influenced by the ischemic duration, inflammatory milieu, and blood–brain barrier integrity. Understanding these differences may help in developing targeted therapeutic strategies to modulate microglial activation and mitigate neuroinflammation in ischemic stroke patients.

The integrity of white matter is of critical importance for brain function. Neuroimaging studies have shown that male WM volume is 456 ± 48 cm^3^ and female WM volume is 392 ± 42 cm^3^, accounting for about 40% of the total volume of the human brain [10]. Cerebral ischemia is highly likely to damage white matter because it is heavily dependent on a continuous supply of oxygen and glucose. In addition, compared with gray matter, white matter has less collateral circulation and blood supply and is extremely sensitive to ischemia. White matter injury (WMI) develops rapidly in ischemia and worsens and peaks 1–3 days after reperfusion [11]. Many studies have shown a linear correlation between the degree of WMI, the size of cerebral infarction, and the National Institutes of Health Stroke Scale (NIHSS). That is, the more severe the degree of WMI is, the more serious the functional impairment will be under the same level of cerebral infarction size [12, 13, 14]. However, the extent and mechanisms of WMI may also vary across stroke subtypes. For example, small vessel disease‐related lacunar infarcts predominantly affect deep white matter tracts, whereas large artery atherosclerosis may disrupt broader cortical–subcortical networks [5]. This subtype‐specific variability in WMI patterns underscores the importance of integrating stroke subtype stratification into investigations of microglial dynamics and remyelination processes. Therefore, it is exceptionally urgent to explore the mechanisms and methods to reduce the damage of white matter and promote the recovery of white matter after stroke [15].

Studies illustrate that demyelinating WMI in stroke primarily results from oligodendrocyte death and myelin lipid metabolism disorder caused by endogenous or exogenous injury [16, 17]. Oligodendrocytes are myelin‐producing cells. The subsequent mature myelin sheaths induced by oligodendrocytes wrap the internodes of axons to facilitate the skipping of nerve impulses. However, oligodendrocytes are very sensitive to ischemia and are prone to ischemic death. Activating microglia in the early cerebral ischemia releases many pro‐inflammatory factors, leading to oligodendrocyte death and aggravating white matter damage [18]. Myelin lipid metabolism disorder is an independent factor leading to demyelination independent of oligodendrocyte death. Ischemic brain injury impairs cholesterol synthesis and transport and reduces myelin lipid levels, resulting in myelin injury.

Microglia, also known as resident macrophages in the brain, are responsible for dynamic monitoring of the environment, regulating neuronal excitability, synaptic connections, neurogenesis, and other functions [19]. In the early stage of ischemic injury, it is activated rapidly and lasts for a long time [20]. A large number of studies have shown that microglia play an important role in the early injury and chronic recovery of cerebral ischemia [21]. When activated, microglia engulf myelin fragments, which contain many lipid components. Therefore, microglia can affect myelin survival and regeneration by influencing myelin metabolism. After ischemic stroke, damaged myelin sheaths and nerves initiate a self‐repair process [22].

It has been reported that remyelination of denuded or newly formed axons occurs in the area surrounding cerebral ischemia and infarction. Remyelination is a process in which oligodendrocyte progenitor cells proliferate, differentiate, mature into functional myelin sheath, and rewrap axons. However, in the process of remyelination, most oligodendrocyte progenitor cells stopped differentiation at the early stage of differentiation into mature oligodendrocyte, and even a small number of proliferating oligodendrocyte progenitor cells differentiated into astrocytes, resulting in incomplete remyelination [23]. After activation, microglia can show different phenotypes depending on the time and area of injury. Recently, many studies have indicated that the classification of microglia into M1 and M2 is an oversimplification, but there is still no uniform consensus classification method. Some studies have found that four clusters of transcription factors regulate microglia phenotypes in rats after ischemic stroke, suggesting that after ischemic stroke, the classification into M1 and M2 cannot be made only in a gross manner [24]. Additional studies have identified 5 microglia types [25], even some studies have shown the existence of 14 microglia subpopulations [26]. The genetic classification of microglia needs further study. So far, the typology and nomenclature of microglia in ischemic stroke are not uniform, but the most studied are still divided into pro‐inflammatory and immune‐regulatory types around their role in inflammation, so this review still uses pro‐inflammatory and immuno‐modulatory types to discuss. Type pro‐inflammatory secretes pro‐inflammatory factors. Contrary to pro‐inflammatory, immune‐regulatory microglia can produce anti‐inflammatory factors, and many studies have proved that immune‐regulatory microglia are beneficial to myelin regeneration [27]. Therefore, this review focuses on the changes and effects of microglia on WMI recovery in ischemic stroke to provide new ideas for reducing stroke WMI and promoting poststroke white matter recovery.

To synthesize current knowledge, this review employed a systematic approach, analyzing peer‐reviewed articles up to December 2024, prioritizing studies with mechanistic insights into microglial roles in white matter recovery. Inclusion criteria focused on experimental models of ischemic stroke, microglial polarization, and remyelination pathways, while excluding non‐English publications and studies lacking histological or molecular evidence.

## 2 Stroke‐Induced Microglial Temporal, Morphology, Phenotype, Functional Changes

Microglia are the innate immune cells of the central nervous system [17]. Under physiological conditions, microglia are typically branched, with small cell bodies and ramified processes, and are called “homeostatic microglia” [16]. Microglia are dynamically changing and are responsible for monitoring the physiological environment of the brain at all times. One of the first events to occur after cerebral ischemia is the activation of microglia, which occurs minutes to hours after the onset of an episode [28]. The activated microglia have a long duration, morphological changes, cell markers, and secretion of inflammatory factors.

First, after microglia are activated, it lasts for a long time. Animal studies have shown that microglia are activated and increased 24 h after ischemia, reach a peak on the 4th day, and gradually decline until 28 days. As time goes by, the activated area of microglia can extend to the area around the ischemic focus [29]. However, many researchers did not agree on when microglia activation increased, continued, and resumed resting state after stroke (Table 1), which may be due to different strains of breeds used stroke model and monitoring period, etc. in the study, resulting in inconsistent results. Clinical studies have found that microglia are activated in the acute, subacute, and convalescent phases of cerebral ischemia. Holfelder and colleagues found that activated microglia/macrophages were significantly increased 24 h after cerebral ischemia in 30 human cerebral ischemic brain tissues [37].

**Table 1:** Temporal variation of microglia after different stroke models.

Organism	Model	Infarction time	Tissue source of extraction/testing method	Type of microglia	Marker	Initial	Peak	Decline to preinjury levels	References
6–8‐week‐old male C57BL/6J (bone marrow chimeric mice by transplanting bone marrow)	MCAO/R	30 min	Infarct area/immunohistochemistry	M	F4/80, CD11b	1 h	24–96 h	28 d	[29]
Wistar rats weighing 150–230 g	MCAO	Permanent	Neocortical and thalamic regions/lectin histochemistry	M	lectin; GSA 1‐B_4_‐HRP	1 d	2 d	14 d	[30]
Bone marrow chimeric mice	MCAO/R	30 min	Striatum	M	Iba1	1 d	14 d		[31]
Male 10–12‐week‐old C57/BL6 mice	MCAO/R	60 min	Cortex and striatum/immunohistochemistry	M1	Inos, CD16, CD32, CD86	1 d	14 d		[20]
				M2	CD296, Arg1, CCL‐22, Ym1/2, IL‐10, TNF‐β	1~3 d	3~5 d	14 d	
Wistar rats (8 weeks old; weighing 260–300 g)	MCAO/R	90 min	In and around peri‐infarct tissue of ischemic brains/immunofluorescence	M	Localization of Iba1 and NG2	NG2^+^ microglia in the demarcation zone and NG2^−^ in the peri‐infarct tissue on Day 7			[32]
Young (3–4 months) and aged (18–20 months) male Sprague‐Dawley rats	MCAO/R	70 min	Infarct area/immunohistochemistry	M	ED1	Young rats: on Days 3 and 7, microglia in the infarct area were gradually activated; on Days 14 and 28, fully activated. Elderly rats: microglia were activated in large quantities on Days 3 and 7; microglia number and activation phenotype decreased on Days 14 and 28.			[33]
Mice (129/SvEv strain) aged 5–9 weeks, both males and females, weighing 18–25 g	Photothrombotic lesion	Permanent	Cerebral cortex and cerebellar sections	M	Iba‐1	On Day 1 after injury, the number of activated microglia increased, and on Day 4, the number of activated microglia further increased, and a high density of activated microglia gathered around the lesion. On Day 7, activated microglia had infiltrated the lesion site and remained infiltrated on Days 14 and 30.			[34]
BALB/c mice, 9 weeks of age	pMCAO (electrocoagulation just proximal to the origin of the olfactory branch)	Permanent	Cerebral cortex/immunohistochemical	M1/M2	CD206 and arginase1/CD16/32/Ym1	Ym1‐positive cells and Arginase1‐positive cells showed increases in WT mice at 24 h; CD206‐positive cells and CD16/32‐positive cells increased in WT mice at 24 h and at 7 days.			[35]
Young male C57BL/6J mice (10‐week‐old); aged male C57BL/6J mice (18‐month‐old)	Distal MCAO model with CCAO	Permanent	Striatum/immunohistochemistry	M1/M2	CD16/Iba‐1; CD206/Iba‐1	Double immunofluorescence staining of CD206 and Iba‐1 revealed a peak in the M2 phenotype 7 d after dMCAO. In contrast, the M1 polarization marker CD16/32 demonstrated a delayed peak at 14 d after dMCAO. Notably, aged animals exhibited a dramatic elevation in the number of CD16/32^+^Iba‐1^+^ M1 microglia/macrophages under sham conditions. This M1 shift was maintained in aged mice until 3 d after dMCAO. In addition, the number of M2 microglia/macrophages was significantly lower in aged mice after dMCAO.			[36]

*Note:* The results of studies on the type of microglia activation and the time to appear and reach the peak after stroke are not consistent. Judging from the table, this may be caused by factors such as the way the experimental model was made, the time of execution, etc.

Abbreviations: M, microglia; MCAO/R, middle cerebral artery occlusion/reperfusion; CCAO, common carotid artery occlusion.

In clinical experimental studies, it was found that activated microglia can be detected in the ischemic zone within 24–48 h of cerebral ischemia. The activation of microglia was observed in six patients, 3–150 days after ischemia. One of the patients was examined on the 28th and 150th day after ischemia. It was found that on the 28th day, the microglia activation was mainly concentrated in the innermost infarct, while on the 150th day, the microglia activation range extended to the connecting area of the ipsilateral and contralateral thalamus [38]. Intriguingly, the activation of microglia in the peri‐infarct area can last up to 14 weeks, which is longer than the activation time in the infarct area, which suggests that the inflammatory response in the area around the infarction lasts longer [38]. The neurons in the ischemic penumbra or peri‐infarct area are considered to be alive and functionally salvageable compared to the core area of injury [39]. Therefore, we should pay more attention to the immune status of the peri‐infarct area to avoid further expansion of the injured area and to promote recovery of the damaged tissue.

**Figure 1:**
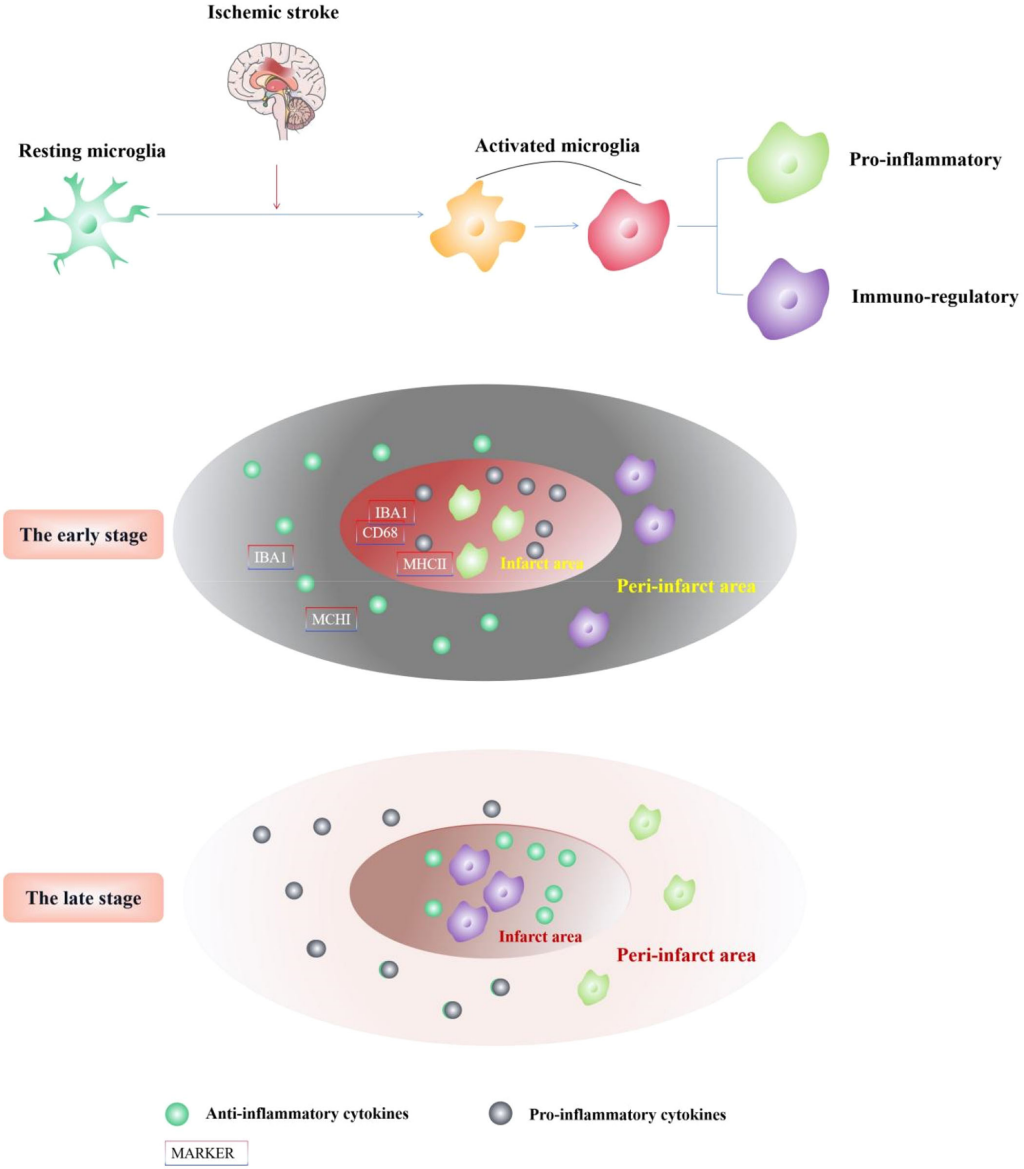
Morphological and temporal changes of microglia after stroke. After stroke, microglia change from a resting state to an activated state. The activation types of microglia have apparent temporal and spatial characteristics. In the early stage of stroke, anti‐inflammatory M2 microglia were highly expressed in the peri‐infarct area, while in the late stage, they were highly expressed in the infarct core area, accompanied by changes in the expression of specific markers and secreted products. In addition, microglia show different expression patterns of markers in a separate area.

Second, the activated microglia will undergo typical morphological changes (Figure 1), manifested explicitly as the contraction and thickening of the protrusions and the hypertrophy of the cell body. One to three days after ischemia, strong and shortened stellate microglia can be seen around the infarct. Six days later, they become amoeboid microglia with large and round cells and no protrusions [40]. At this time, other studies have reported that the expression of P2y12 is reduced when microglia are activated in an amoeboid round shape after inflammatory stimulation [25]. This is consistent with the results of genetic changes in microglia after stroke discussed in our previous sections. The amoeboid microglia are more conducive to the high‐speed movement of the microglia and reach the injured site quickly within a few minutes [41]. A 2021 review summarized the temporal changes in microglia activation and morphology after permanent middle cerebral artery occlusion (pMCAO) [42]. Twenty‐four hours after pMCAO, microglia were activated in cortical and thalamic regions. At 24~72 h after pMCAO, microglia near the injury were star‐shaped with thick and short protrusions, whereas at 6 d after pMCAO, microglia were amoeboid in the same area. Due to advances in imaging techniques, Rupalla et al. [43] found microglia activation as soon as 30 min after pMCAO, showing hypertrophy of the cytosol and protrusions within the penumbral band. In recent years, a series of studies have identified activated microglia in the acute [34], subacute [44], and chronic [35] phases of stroke. The morphological changes of microglia may be related to the function of receptors and the complex secretory capacity of cells. Some studies have shown that the expression of the purinergic P2Y12 receptor (P2Y12) is related to microglia's activation, migration, and morphological changes [45]. Adipose‐tissue‐derived mesenchymal stem cells (ASCs) secrete colony‐stimulating factor‐1, which also changes microglia's morphology and expression level [46].

In addition, microglia show different expression patterns of markers in a separate area (Figure 1). In the infarct zone, both IBA1 and CD68 (ED‐1) in microglia are positive, and the expression of CD68 is enhanced, while in the peri‐infarct region, microglia are positive for IBA1 and negative for CD68 [47]. Studies have also found that activated microglia in the infarct core area mainly express MHC I and are responsible for clearing cells, while the activated microglia in the penumbra mainly express MHC II, which may be related to Wallerian (anterograde) deformation [42, 48]. Differences in these markers suggest distinct microglial phenotypes in the injured core and surrounding areas. Hu and colleagues found that in the transient focal cerebral ischemia model, the polarization of microglia is a dynamic process of change. After a stroke occurs, a large number of microglia migrate and infiltrate the infarct. At this time, the microglia are of immunoregulatory phenotype in the peri‐infarct area, which provides endogenous support for eliminating dead cells and restricting the further expansion of the brain injury area. However, microglia gradually decreased within 7 days, and pro‐inflammatory microglia accounted for the primary injury area [20]. Other analogous studies have shown that the early microglia are immunoregulatory phenotype, a protective type, while the later stage transitions to the type associated with axons damage [49]. The above phenomenon suggests that the continued presence of pro‐inflammatory microglia will affect the prognosis of stroke, so inhibition of pro‐inflammatory microglia or promotion of pro‐inflammatory to immuno‐regulatory transformation will be beneficial to the recovery of stroke. Pro‐inflammatory microglia were positively correlated with disease severity, with maximum expression at 7 d poststroke. Anti‐inflammatory microglia expression, which was not correlated with disease severity, increased at 11–13 days poststroke [50]. This speculation has now been confirmed by studies. MiRNA‐124 protects neurology and promotes functional improvement by inverting the microglia phenotype after focal cerebral ischemia [51]. However, there is still a gap in the validation of clinical studies.

The activated microglia also secrete a variety of mediators. The effects of these mediators on cerebral ischemic tissues are complex, and they have the dual impact of aggravating or reducing cerebral ischemic lesions. After cerebral ischemia injury, microglia will quickly join the aggressive inflammatory response, release pro‐inflammatory factors and neurotoxic molecules, such as IL‐1β, TNF‐α, and ROS, and recruit more immune cells after activated microglia [52]. On the other hand, microglia also release anti‐inflammatory factors, such as IL‐4, IL‐10, etc., to produce anti‐inflammatory effects and participate in repairing inflammation after brain injury [52]. The pro‐inflammatory/anti‐inflammatory effects of microglia after stroke change dynamically with changes in the microenvironment in the brain. In a mouse stroke model, it was found that in the early stages of ischemic stroke, the predominant microglia type in the ischemic area is the anti‐inflammatory type. After a few weeks, the primary phenotype was pro‐inflammatory [40]. The current consensus is that severe inflammation in the acute phase is negatively correlated with the prognosis of stroke [53].

In conclusion, the activation time of activated microglia after cerebral ischemia is early and lasts a long time. In addition, microglia activation types, markers, and secretory factors are time‐specific and region‐specific, suggesting that the role of microglia in poststroke white matter pathological analysis should be time‐specific and region‐specific.

## 3 Advances in the Mechanisms by Which Microglia Impact Myelin Regeneration

During the development of the central nervous system, microglia play an important role in regulating OPC homeostasis. As a subtype of glial cells, microglia have significant interactions with another kind of glial cells, oligodendrocytes. White matter damage caused by brain injury is characterized by demyelination injury [54]. Based on previous studies, microglia are closely related to the white matter damage. Olah and colleagues conducted a genome‐wide gene expression analysis of corpus callosum microglia during demyelination and remyelination of copper ketone model mice. The results showed that there are differentially expressed genes (DEGs) in microglia and the following processes that affect myelination related: (1) Lrp1, Calr, Cd14, Itgb2, Itgam, Lgals3, etc., involving in phagocytosis of apoptotic and myelin fragments; (2) recruit oligodendrocytes and provide nutritional support for them, such as Cxcl10, Cxcl13 and Igf1, Tgfb1, Pdgfa, and Pdgfb; (3) participate in organizational remodelings, such as Mmp12 and Mmp14 [55]. These studies suggest that microglia are closely related to remyelination. In one of the vivo studies, one study of permanent focal cerebral ischemia observed that microglia activation increased 2–4 weeks after MCAO, while oligodendrocyte loss increased [56]. And in one of the vitro studies, a previous study presented that OPCs differentiation and myelination significantly increased compared to the control and astrocyte culture medium groups when OPCs were cultured in a microglial cell‐conditioned medium [57]. Recent findings show that CD11c+ microglia increase after ischemic stroke and promote white matter repair [58].

### 3.1 Reasons for Impaired White Matter Repair After Stroke: Inflammation

A clinical study shows that inflammation leads to reduced white matter volume and/or disrupts white matter integrity [59]. The immunity plays an important role in brain WMI and repair after stroke [60]. Following cerebral ischemia, both innate and adaptive immune cells are activated. Moderate immune responses are beneficial in reducing brain injury and promoting recovery, however, excessive or unselective immune responses can have the opposite effect, exacerbating brain injury and impeding brain repair [61]. Numerous sequencing studies of tissue samples have also found that some of the DEGs in microglia from both groups of samples were associated with inflammatory responses in both the stroke model and sham‐operated model samples. Using single‐cell RNA sequencing (scRNAseq) to study brain‐infiltrating immune cells in the chronic phase of poststroke mice, the investigators found that microglia predominate in cells at 5 days poststroke based on enriched genes per cluster, consistent with a predominant innate immune response in the early poststroke period [61]. In another experiment, scRNAseq was performed on the sham‐operated and MCAO groups, and there were 274 differential genes, including 157 unique DEGs associated with microglia. Among them, microglia and cell adhesion molecules shared the most commonly DEGs, and both microglia and cell adhesion molecules were associated with neuroinflammation. This suggests that microglia are involved in regulating stroke‐induced neuroinflammation after cerebral ischemia. In addition, differentially expressed microglia clusters contain monocyte chemotactic protein (MCP) family genes such as CCL12, CCL2, and CCL7. MCP family genes play a key role in the pro‐inflammatory response through the chemotaxis of monocyte‐derived macrophages and other peripheral immune cells to sites of ischemia [25]. P2y12R, the P2 purinoceptor gene, is a marker gene for microglia and is specifically downregulated in microglia after MCAO. Previous studies have reported that P2y12R expression is similarly reduced in the inflammatory environment following microglia activation [62].

Microglia express multiple ionotropic (P2X) and metabotropic (P2Y) purinergic receptor subtypes that act as injury sensors and trigger a robust microglial inflammatory response, which could have detrimental role (e.g., P2Y12) or favorable role (e.g., P2X7) [63]. So the activation state of microglia varies according to the change of physiological environment, and there are two classic activation types: pro‐inflammatory and immune‐regulatory [64]. Additionally, microglia subtypes secrete cytokines, and it is reported that the secreted cytokines will have a direct impact on remyelination [20]. The two activation types have opposite effects on remyelination. Studies have shown that the conversion of microglia pro‐inflammatory to immune‐regulatory phenotype is related to remyelination [65].

The traditional view is that pro‐inflammatory microglia secrete pro‐inflammatory factors in the early stage of brain injury, initiate and spread the inflammatory cascade, and cause white matter demyelination damage. Pro‐inflammatory microglia secrete IL‐1β, TNF‐α, IL‐6, IFN‐γ, and other cytokines [66]. Extensive studies have shown that the cytokines TNF‐α and interleukin IL‐1β secreted by pro‐inflammatory microglia have cytotoxic effects on oligodendrocytes. In animal studies, neonatal mice treated with IL‐1β showed marked hypomyelination with cognitive deficits at P35 [67]. The impacts of IL‐1β on white matter are similar in other disease models. Inhibition of IL‐1β with IL‐1β antagonist showed a decrease in LPS‐induced white matter damage and oligodendrocyte death, as well as an improvement in EEG (electroencephalograph activity) in fetal sheep [68]. Double staining of tMCAO rat brain sections with TNF‐α and Iba1 showed that TNF‐α was mainly expressed on microglia, increasing from 12 h and reaching its highest level on Day 3 after tMCAO [69]. Pasquini and colleagues found an increase in oxidative stress products in oligodendrocytes treated with TNF‐α [70]. Another study also found that microglia activate and express TNF‐α at the WMI, and oligodendrocytes die in the form of caspase3‐dependent apoptosis [71].

However, studies have shown that IL‐1β and TNF‐α are beneficial to remyelination. The coculture of neurospheres and microglia can enhance the differentiation of NSCs into OPCs, by inhibiting the production of cytokines such as IL‐1β by microglia can prevent their differentiation [66]. In the IL‐1β^−/−^ demyelinating mouse model, it was found that mature oligodendrocytes and remyelination decreased, suggesting that IL‐1β plays a crucial role in remyelination, and it was also found in IL‐1β mice, the expression of IGF‐1 is reduced, and it is speculated that IL‐1β may regulate myelination through IGF‐1 [72]. Arnett and colleagues used a mouse demyelination model lacking TNF‐α and its receptors and found that remyelination was significantly delayed and mature oligodendrocytes were reduced. Among them, TNFR‐1 is more related to oligodendrocytes [73]. Correspondingly, Wang and colleagues found that in vitro, the use of histone deacetylase (HDAC) inhibitor scriptaid through GSK3β/PTEN/Akt axis promoted the transition from pro‐inflammatory to immunoregulatory, resulting in microglia and macrophages producing fewer TNF‐α and NO increases the preservation of cocultured oligodendrocytes, and in vivo experiments have also been found to reduce white matter damage [74]. In summary, most studies prove that the inflammatory factors produced by pro‐inflammatory microglia are not conducive to myelin regeneration. Still, we should not blindly deny or ignore some studies with opposite conclusions that inflammatory factors secreted by microglia benefit myelination, suggesting that we need to further explore the role of pro‐inflammatory type microglia in myelin regeneration.

Immunoregulatory microglia are protective cells that are anti‐inflammatory and polarized and can promote the differentiation of oligodendrocytes during the remyelination process after a stroke. Studies have shown that the coculture of immune‐regulatory microglia conditioned medium with oligodendrocytes enhances the differentiation of oligodendrocytes, while in the medium lacking immune‐regulatory microglia, oligodendrocytes differentiate weaken [75]. Recently, the increasing evidence shows immune‐regulatory microglia can secrete anti‐inflammatory and neuroprotective cytokines, such as IGF‐1, BDNF, and PDGF‐α, promoting the differentiation of hippocampal neural progenitor cells into oligodendrocytes and preventing immature oligodendrocytes from apoptosis [27]. It has been summarized in detail about cytokines to myelination in my recently published review [76].

In addition to affecting the differentiation of OPCs, immune‐regulatory microglia can also affect the proliferation of OPCs. Studies have shown that vascular endothelial growth factor (VEGF‐C) produced by microglia after ischemia can stimulate the proliferation of OPCs through the VEGFR‐3 receptor [77].

### 3.2 Reasons for Impaired White Matter Repair After Stroke: Phagocytosis

Phagocytosis of microglia is important for effective myelin regeneration. The clearance of myelin debris after myelin injury affects OPC recruitment, differentiation, and remyelination. The number of microglia is significantly higher in the white matter of the normal human brain than in the gray matter, and the same is true in the adult rat brain [78]. In addition to the difference in numbers, microglia in white matter have higher phagocytic activity. Microglia are involved in the internalization of myelin debris, phagocytosis, lysosome maturation, and cholesterol recirculation. It was found that microglia in white matter expressed more phagocytosis‐related proteins, such as CD68 and CD86, than in gray matter [79]. Some related studies have further investigated the mechanism of the above phenomenon. Huizinga et al. coincubated gray matter and white matter from human with human microglia and found that the number of microglia containing myelin was significantly higher in microglia incubated with white matter, a finding that suggests that OPC in white matter may be a white‐matter‐specific factor that promotes phagocytic activity of microglia [58]. The above findings suggest that phagocytosis of microglia has a more important role in the recovery from WMI than gray matter.

There have been several studies on the mechanisms related to microglia phagocytosis after ischemic stroke. Lpl and CD72 in microglia are upregulated after 24 h of MCAO, considering that Lpl and CD72 may be involved in the clearance and recycling of lipid debris [80]. Several receptors expressed by microglia and regulating myelin debris clearance and catabolism have now been identified.

1. CX3CR1
  The first is CX3CR1, which was found to be highly expressed in microglia [81]. Full knockout of Cx3cr1 in mice revealed that post‐demyelinating phagocytes lacked internalized myelin and endosomes, suggesting an impaired ability to take up myelin fragments [82].
2. TREM2
  Myelin is rich in lipids, and myelin regeneration is closely related to lipid homeostasis [83]. Cholesterol is one of the imperative lipid components. During brain development, oligodendrocytes synthesize most of the cholesterol to promote the expansion of the myelin membrane. Accordingly, oligodendrocyte cholesterol synthesis has a limited rate of myelination [84]. When remyelination, oligodendrocytes reduce transcripts of cholesterol synthesis. Myelin debris contains plenty of cholesterol. Microglia/macrophages can absorb the cholesterol when consuming myelin debris. The intake of cholesterol will be an important source of raw materials for oligodendrocytes to form myelin [85]. Immunoregulatory microglia can remove damaged myelin fragments, maintain the stability of myelin lipid metabolism, and ensure sufficient materials for myelin lipid synthesis from remyelination after ischemic stroke. Studies have shown that in the photothrombotic cerebral ischemia model, microglia play a major role in phagocytosis within 3 days after cerebral ischemia.
  The phagocytic function of microglia is mainly related to the TREM2 receptor on the cell. In mice subjected to MCAO, TREM2 is highly expressed on microglia, but not on astrocytes, oligodendrocytes, and neurons [86]. One more study, including in vitro and in vivo studies, also confirmed the same results. Both transcriptional and posttranscriptional levels of TREM2 in the ischemic penumbra and cultured primary microglia were increased after occlusion and reperfusion of the middle cerebral artery occlusion in mice in vivo and after oxygen–glucose deprivation and reoxygenation of primary microglia in vitro [86].
  The main component of myelin, phospholipid, is one of the ligands of TREM2. TREM2 can recognize this ligand, remove damaged myelin fragments, and release growth factors to assist oligodendrocyte remyelination [87]. Studies have found that in the absence of TREM2 microglia cannot sense myelin damage and cannot further initiate appropriate clearance and regeneration procedures. The overexpression of TREM2 in the experimental autoimmune encephalomyelitis mouse model found that the severity of demyelination was significantly reduced [88]. In an ischemic stroke model, Nhe1 cKO microglia showed similar upregulation of the TREM2 gene, along with activation of the LXR/RXR pathway and enhanced phagocytic activity [89]. Kawabori and colleagues found in vitro that knocking down microglial TREM2 activated microglia into an amoeba phenotype, reducing their phagocytosis of damaged neurons. In vivo, compared with wild‐type rats, TREM2 knockout mice showed reduced numbers of activated microglia and phagocytes, almost absence of foam macrophages, reduced absorption of infarcted brain tissue, deterioration of ipsilateral hemispheric nerve recovery, and increased brain tissue damage. Trem2‐FC fusion protein, which is used to identify potential TREM2‐binding partners, is colocalized to neurons by binding TREM2 ligands. Oxygen–glucose deprivation‐exposed neuronal media or cellular components containing nuclear or purified DNA stimulate signal transduction through TREM2. The TREM2‐FC fusion protein reduces nucleic acid in ischemic brain lysates. Nucleic acid may be a potential ligand of TREM2 in cerebral ischemia [90]. TREM2 is also related to inflammation. Studies have found that manipulating the expression level of TREM2 in vivo and in vitro can regulate the production of inflammatory mediators after ischemia. Overexpression of TREM2 significantly inhibited the inflammatory response, while silencing TREM2 enhanced the inflammatory response and aggravated the degree of nerve damage [86]. The timely removal of cells and myelin debris after stroke is conducive to the recovery of white matter damage. TREM2 is closely related to the phagocytic function of microglia [90]. In clinical studies, TREM2 is cleaved to myeloid soluble trigger receptor 2 (sTREM2) in plasma extracted from patients with ischemic stroke within 24 h, results of which suggest that early increase in sTREM2 levels is a predictor of poor outcome in stroke [91]. Promoting the expression of TREM2 may become one of the important directions for reducing stroke brain damage and improving prognosis in the future.
3. TLRs
  Microglia have two different types of phagocytic receptors. One type of receptor is pattern recognizing receptor (PRR) that recognizes microbes such as microglia membrane and the other is toll‐like receptor (TLR) that clears pathogenic bacteria and produces pro‐inflammatory factors that lead to an inflammatory response. TLR plays an important role in orchestrating the inflammation of microglia. TLRs are receptors recognizing microbes, one of the phagocytic receptors of microglia that support pathogen clearance while stimulating a pro‐inflammatory response of phagocytes [92]. TLRs recognize pathogen‐associated molecular patterns (PAMPs) expressed in microorganisms. Nod‐like receptors (NLR) are oligomeric cytoplasmic receptors that form inflammasome together with bridging proteins. TLRs and inflammasome receptors act synergistically. Ligand binding to TLRs induces the expression of pro‐IL‐1β and pro‐IL‐18, after which NLR pathway activation regulates their protein hydrolytic processing and release.
  A large number of studies have demonstrated that TLR can mediate polarization of microglia [93, 94]. TLR2 is closely related to microglial inflammation. TLR2 plays an important role in host defense against pathogens and in the response to noninfectious inflammation. Microglial TLR2 mediates pathological processes in many neurological diseases. Alzheimer's‐related research has found that early mild stimulation of microglial TLR2 receptors attenuates cognitive dysfunction by modulating the inflammatory and anti‐inflammatory phenotypes of microglia [95]. It has been shown that TLR2 pathway signaling is diminished after cerebral ischemia, and it has also been found that TLR2 is minimally reduced in older brains, which may inhibit M2‐like responses and thus allow for less brain remodeling and recovery after stroke [24]. Studies have found that compared with WT mice, ROS in mice after TLR2 gene deletion is reduced, suggesting that TLR2 can promote the inflammatory response of microglial cells and aggravate inflammatory damage [96]. One study has found that in the chronic hypoperfusion brain injury model, TLR4‐dependent autophagy can promote the pro‐inflammatory polarization state of microglia through the STAT1/6 pathway, thereby causing white matter damage [97]. One more study showed that the HSP60 released by the activated pro‐inflammatory type microglia induced by LPS could cause OPC apoptosis after binding to OPC. HSP60 is the endogenous ligand of the TLR4 complex. After pretreatment with blocking TLR‐4 complex, the activity of OPC increased, and the expression of NF‐κB decreased. This result suggests that HSP60 released by microglia may bind to TLR4 to activate the TLR4–NF‐κB signaling pathway to mediate OPC apoptosis [98]. Also, during cerebral ischemia, necrotic nerve cells release dangerous molecules, for example, high‐mobility group box 1 (HMGB1), extracellular peroxiredoxin (Prx) family proteins, Galectin‐3 (Gal3), etc. These molecules are collectively termed damage‐associated molecular patterns (DAMPs). When stimulated by DAMPs, TLRs activate the NF‐κB pathway and induce the release of pro‐inflammatory microglia inflammatory factors. NF‐κB is closely related to neuroinflammation caused by microglia and can control the production of cytokines and cell survival, thereby affecting the degree of white matter damage [99].
  The regulation of TLRs on microglia inflammatory response cannot be ignored, which will become an important therapeutic target for regulating central microglia‐derived inflammatory injury.
4. phosphatidylserine (PS), Lpl, and CD72.
  The other type of phagocytic receptors is receptors recognizing apoptotic cellular material such as receptors that recognize phosphatidylserine (PS), which phagocytose apoptotic cell cadavers but also produce an anti‐inflammatory response [100]. Studies have shown that after myelin injury, microglia express genes related to phagocytosis and lysosomal pathways [55]. The ability of microglia/macrophages to phagocytose myelin debris is also impacted by age factors; the older the age, the lower the ability to clear myelin debris, and this reduced ability correlates with a reduced efficiency of myelin regeneration [101].
  There have been several studies on the mechanisms related to microglia phagocytosis after ischemic stroke. Lpl and CD72 in microglia are upregulated after 24 h of MCAO, considering that Lpl and CD72 may be involved in the clearance and recycling of lipid debris [80]. Several receptors expressed by microglia and regulating myelin debris clearance and catabolism have now been identified.
  In summary, these findings demonstrate the existence of a complex regulatory network in which several receptors coordinate microglia clearance of debris after injury and induce the expression of additional receptors and enzymes that facilitate this process.

### 3.3 Brain‐Derived Microparticles (BDMP) and Myelin

The microparticles are derived from intact cells and are membrane‐bound vesicles shed from the cell membrane, ranging in size from 0.1 to 1 mm [102]. According to their sources, these MPs can be divided into two categories: one is derived from endothelial cells, platelets, white blood cells, etc., and released in the circulating blood; the other is brain‐derived MPs [46]. MP contains antigens from mother cells and participates in the communication between cells and the regulation of inflammation [103]. After a stroke or other brain damage, the cells are activated and release massive MP into the blood and body fluids. Circulating MPs have been studied as a potential biomarker for neurological diseases such as ischemic cerebral infarction and transient ischemic attack [104]. Studies have found that after a stroke, the secretion of BDMP increased, which in turn activates microglia/macrophages and triggers neuroinflammation [105]. Researchers used lactectin to inhibit BDMP from activating microglia to cause inflammation, thereby reducing the damage of inflammation to the brain's white matter structure [106]. The above studies suggest that by increasing the clearance of BDMP, inflammation caused by microglia can be reduced, and white matter damage caused by inflammation after stroke can be alleviated.

### 3.4 Proteolytic Enzymes Matrix Metalloproteinase (MMP) and Myelin

Matrix metalloproteinases (MMPs) are calcium‐ and zinc‐dependent enzymes that degrade extracellular matrix proteins, such as collagen, as part of the enzyme cascade [107]. There is evidence that MMP‐9 expression increases in the infarct and surrounding penumbra areas within 2 h after ischemic and hemorrhagic stroke. MMP‐9 knockout mice have more minor brain damage than wild‐type mice [108]. Another study suggested a strong relationship between MMP and microglia, inasmuch as the study found MMP3 and MMP9 are highly expressed on microglia/macrophages 72 h after ischemia [109]. When LPS‐induced microglia were activated, it was found that activated microglia produced MMP‐9 [110]. Furthermore, in the cerebral ischemia‐reperfusion model, neuroinflammation mediated by MMP‐3 and MMP9 is important for white matter damage and oligodendrocyte apoptosis [18]. MMP3 is an endogenous neuroactivator of microglia, activating microglia to release inflammatory factors [111]. The autopsy studies showed that MMP3 and MMP9 were highly expressed in white matter damage after stroke [112, 113]. After knocking out the tissue inhibitor of metalloproteinases‐3 (TIMP‐3) in mice, it can reduce the expression of MMP3 and MMP9 and reduce the death of immature oligodendrocytes after cerebral ischemia [109]. And another animal study showed that inducible MMPs, such as MMP‐9 and MMP‐3, can cause brain white matter damage for hours or days [114]. In vitro experiments, studies have also confirmed that MMP affects myelination [115]. The study results show that MMP‐2 is the main enzyme that degrades myelin basic protein (MBP), followed by MMP‐3 and MMP‐9. After inhibiting MMP, glial cell activation and MBP degradation are both reduced [116]. In conclusion, MMP is closely related to microglia‐mediated inflammatory damage and is involved in WMI and myelin regeneration after stroke.

### 3.5 Astrocyte Affects Microglia During Remyelination

In stroke damage, the activation of astrocytes is later than that of microglia. Microglia can promote the activation of astrocytes, while astrocytes, in turn, affect the migration and inflammatory response of microglia. Microglia can express an assortment of purinergic receptors, such as P2X4/P2Y12. It is reported that in laser‐induced injury, neuronal *N*‐methyl‐d‐aspartate receptor (NMDAR) was activated and triggers astrocytes to release ATP [117], which activates microglia P2Y12 [118]. In addition, another study found that TNF‐α stimulated astrocytes to enhance the release of S100B in brain injury [119]. High concentrations of S100B bind to receptors for advanced glycation end products and participate in the regulation of microglia activation [120]. ATP and S100B may both be messengers and communication mediators between astrocytes and microglia in stroke injury [121]. It has also been reported that astrocytes can release GABA and trigger microglia transcription factor Nrf2 to weaken the inflammatory response of microglia [122, 123]. Astrocytes also affect the phagocytosis of microglia by releasing milk globulin epidermal growth factor 8 (MFGE8), which binds to phosphatidylserine on apoptotic cells as a marker for microglia to recognize apoptotic cells, and then to perform apoptotic phagocytosis [124]. These findings suggest that astrocytes affect the activation of inflammatory responses and phagocytosis of microglia, and that the functional status of these microglia will affect myelin regeneration.

### 3.6 Other Mechanisms Concerning Microglia Influence Remyelination

Some studies have further explored the mechanism by which microglia affect myelin regeneration. The activation of the Na^+^/H^+^ exchanger (NHE1) is necessary for a variety of microglia functions. The researchers knocked out the Nhe1 gene on microglia and found increased myelin production in white matter 14 days after stroke compared with prototype mice [125]. Furthermore, excitotoxicity caused by glutamate is a major destructive cause of stroke and other forms of cerebral ischemia [126]. Microglia express cysteine/glutamate transporters that release glutamate in response to oxidative stress and are therefore also closely associated with glutamate excitotoxicity [127]. Oxidative stress and activation of microglia induced by cerebral ischemia and hypoxia impair the glutamate transporter function of microglia and enhance glutamate release through cysteine/glutamate antiporter (xCT) [64, 66, 70, 72, 73, 74, 128]. Over and above activated microglia, homeostatic microglia can also promote myelin regeneration. Converging studies have shown that inactive microglia can promote the survival and maturation of OPCs. The researchers cultured OPCs in homeostatic microglia conditioned medium and found that the synthesis of myelin‐specific proteins in OPCs increased [129]. Nicholas and colleagues found that homeostatic microglia can act on PDGF‐α receptors through phosphatidyl‐3‐inositol (PI‐3) kinase and synergically increase the activation of NF‐κB with endogenous PDGF‐A chain, thereby producing soluble factors to promote oligodendrocyte development [129].

## 4 Microglial Mechanisms in White Matter Repair Following Ischemic Stroke: Divergent Roles in Hematological Versus Nonhematological Etiologies

Ischemic stroke secondary to hematological disorders exhibits distinct pathophysiological features compared to cerebral ischemia of nonhematological origin, which may drive divergent microglial responses during WMI recovery. Although microglia universally execute core functions—such as tissue surveillance, phagocytic clearance, and trophic factor secretion—across ischemic contexts, microenvironmental variations stemming from underlying disease mechanisms critically influence their functional dynamics [58, 130]. In hematological disorders (e.g., leukemia, lymphoma, myeloproliferative diseases), cerebral infarction often arises from hematologic aberrations such as dysregulated coagulation, platelet dysfunction, or compromised vascular integrity. These etiological factors may establish a unique inflammatory milieu that modulates microglial activation states and polarization patterns, ultimately shaping their reparative capacity in white matter regions [78]. Emerging evidence suggests that microglial behavior in hematology‐associated ischemia may diverge from nonhematological stroke models in several key aspects.

The activation trajectory of microglia in hematological disorders may differ due to disease‐specific cytokine profiles and cellular stressors, potentially altering their transition between pro‐inflammatory and reparative phenotypes [58, 78]. Such heterogeneity in activation could further impact their functional outputs, including phagocytic efficiency. For instance, impaired clearance of myelin debris—a critical step for initiating remyelination—may arise from hematopoietic dysfunction or systemic inflammation in hematological disorders, compromising microglia‐mediated repair processes [58, 130]. Additionally, the secretion of oligodendrocyte‐promoting factors (e.g., BDNF, IGF‐1) by microglia might be differentially regulated in these contexts, potentially delaying oligodendrocyte precursor cell maturation and axonal rewiring [78]. The immune crosstalk in hematology‐driven ischemia may also involve heightened interactions between microglia and adaptive immune cells, such as leukemia‐derived lymphocytes, introducing novel immunomodulatory challenges absent in conventional stroke models [130]. Furthermore, sexual dimorphism in microglial responses, previously implicated in stroke outcomes, could be amplified in hematological disorders where perturbations to the hormonal–hematopoietic axis may further polarize microglial function [131].

Despite the biological plausibility of these mechanistic divergences, direct evidence delineating microglia‐specific contributions to WMI recovery in hematology‐associated stroke remains sparse. The interplay between hematopoietic pathophysiology and neuroimmune signaling warrants systematic investigation, particularly regarding the temporal dynamics of microglial phenotype switching and their functional consequences on white matter tract integrity. Elucidating these etiologically distinct mechanisms could inform targeted therapeutic strategies. For example, interventions enhancing microglial phagocytosis or optimizing polarization balance might show differential efficacy across stroke subtypes. Future studies employing etiology‐stratified preclinical models and single‐cell transcriptomic profiling will be essential to unravel these complex microglial adaptations [58, 78], ultimately paving the way for precision immunomodulatory approaches to improve recovery in this clinically distinct stroke population.

## 5 Promoting Microglia Pro‐Inflammatory–Immunoregulatory Transformation Is Beneficial to Remyelination

There is definitive evidence that promoting microglia pro‐inflammatory–immunoregulatory transformation is beneficial to remyelination. It was found that the drug fingolimod can promote the polarization of microglia from pro‐inflammatory to immunoregulatory through the STAT3 pathway, thereby reducing the inflammatory response caused by cerebral ischemia and hypoxia, reducing myelin damage [132]. In vitro studies have also shown that the phenotype of microglia affects the survival of oligodendrocytes. After 24 h of OGD treatment, pro‐inflammatory‐type microglia induced by LPS‐conditioned medium, untreated microglia, and immunoregulatory‐type microglia induced by IL‐4 conditioned medium were cocultured with oligodendrocytes, respectively. It was found that the apoptosis of immunoregulatory‐type microglia decreased, while that of the other two groups increased [133]. A mouse trauma model showed that Scriptaid, a HDAC inhibitor, may protect oligodendrocyte lineage cells in vivo and in vitro by promoting the conversion of microglia from microglia pro‐inflammatory to immunoregulatory. Similar studies have shown similar results [134]. Peroxisome proliferator‐activated receptor‐γ stimulant siglitazone can reduce pro‐inflammatory type microglia, increase immunoregulatory microglia expression, and improve long‐term white matter integrity after cerebral ischemia [135]. In addition to the above methods to promote microglia transformation, other treatments like low temperature, dimethyl fumarate have also been shown to be beneficial for myelin regeneration [136, 137].

The above studies have shown that the changes in the microglia phenotype caused by stroke cause myelin sheath damage, and adjusting the phenotype can reduce the damage of white matter.

## 6 Conclusion

**Figure 2:**
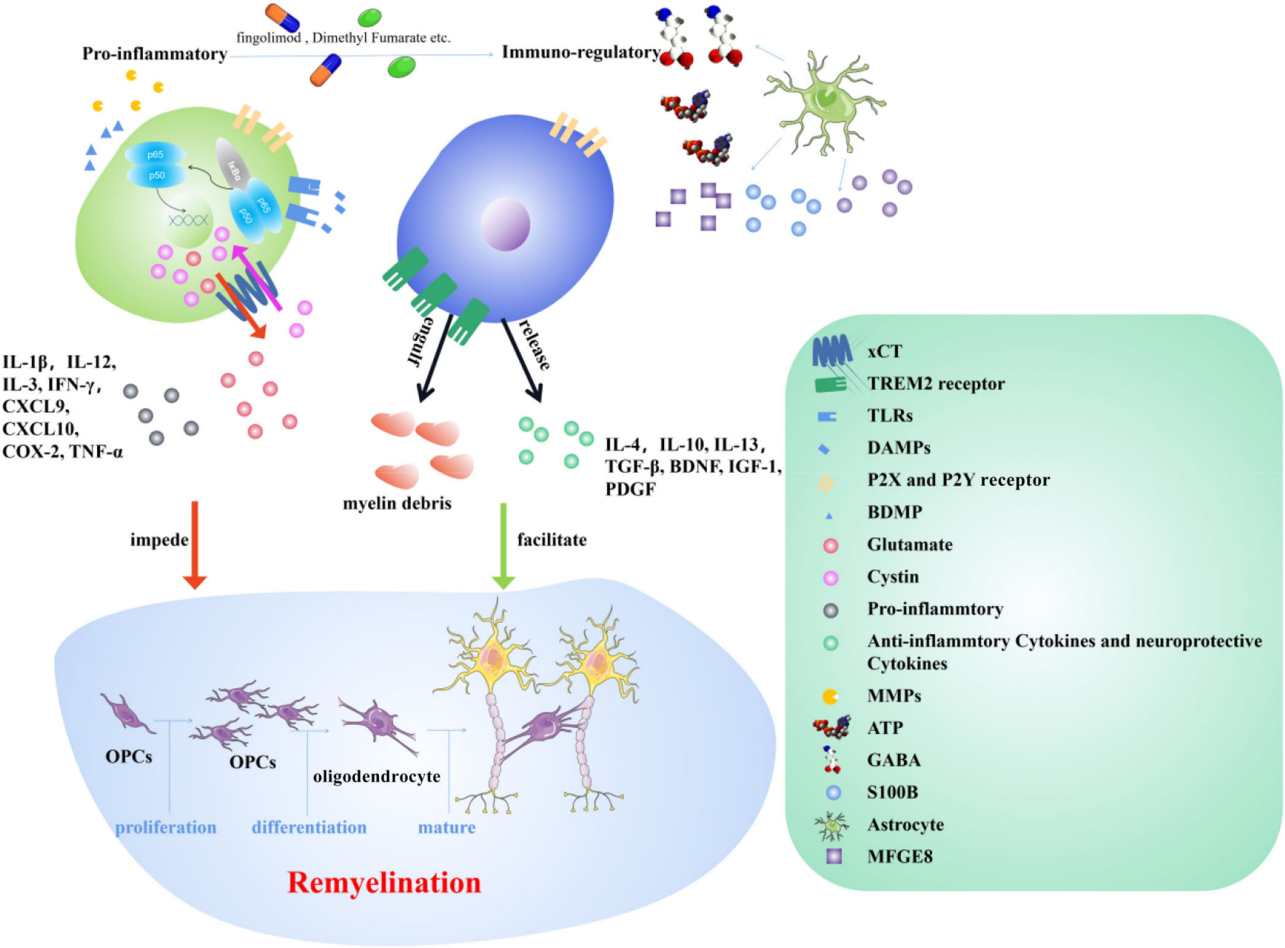
Effect and mechanism of microglia on myelin regeneration. Activated microglia were divided into pro‐inflammatory type M1 and anti‐inflammatory type M2. Microglia in both activated states had distinct effects on myelin regeneration. M1 microglia impair myelin production mainly by releasing pro‐inflammatory cytokines and excitatory toxicity caused by abnormal glutamate metabolism. M2 microglia promote myelin formation mainly through its phagocytosis and release of anti‐inflammatory factors and neuroprotective factors. Astrocytes influence the inflammatory response and phagocytic function of microglia. However, studies on the effect of cytokines released by some M1 microglia on myelin generation are inconsistent, and the specific reasons remain further studied. In conclusion, studies that can promote the transformation from M1 to M2 through drugs have proved beneficial to myelin regeneration, which will become one of the potential drug target directions for white matter recovery after stroke.

In ischemic stroke, microglia are activated in various forms due to different internal environmental states, and the activation state lasts for a long time. For example, microglia activation types differ in early and late cerebral ischemia and central and peripheral areas of injury. In addition, cytokines secreted by microglia with different activation types can be divided into anti‐inflammatory and pro‐inflammatory types. Moreover, more types of microglia are now being discovered. This suggests that the role of microglia in WMI and recovery of stroke should not be simply classified as beneficial or harmful, but should be comprehensively analyzed according to activation type, activation time, region, and secretory factors. This article reviews the changes of microglia in stroke and their effects on myelin injury and myelin regeneration (Figure 2).

While this review synthesizes current knowledge on microglial mechanisms in poststroke white matter recovery, several limitations should be acknowledged. First, the heterogeneity of experimental models (e.g., permanent vs*.* transient ischemia, varying animal strains) and inconsistent temporal monitoring across studies may limit the generalizability of conclusions. Second, the reliance on preclinical data highlights a critical gap in clinical validation, particularly regarding microglial phenotype dynamics in human stroke patients. Third, the conventional M1/M2 classification oversimplifies microglial diversity, as emerging single‐cell studies reveal nuanced subpopulations with distinct functional roles, which were not fully addressed here. Fourth, the complex interplay between microglia and other CNS cells (e.g., astrocytes, oligodendrocytes) in the ischemic microenvironment remains incompletely elucidated. Finally, while therapeutic strategies targeting microglial polarization are promising, their translational feasibility—including safety, specificity, and efficacy in diverse stroke subtypes—requires rigorous investigation.

Future studies should prioritize resolving the spatiotemporal heterogeneity of microglial responses using advanced techniques such as scRNAseq and spatial transcriptomics. This will refine phenotype classifications and uncover subtype‐specific mechanisms. Longitudinal clinical studies integrating neuroimaging and molecular profiling are essential to validate preclinical findings and identify biomarkers for microglial activity in humans. Additionally, interdisciplinary approaches combining metabolomics, epigenetics, and immune modulation could unravel how microglia interact with oligodendrocyte precursors and myelin debris under varying metabolic or inflammatory conditions. Investigating sex‐specific and age‐related differences in microglial function may inform personalized therapies. Finally, developing targeted interventions to enhance phagocytic clearance, optimize polarization balance, or modulate microglia–astrocyte crosstalk holds promise for improving white matter repair and functional recovery in stroke patients. Addressing these gaps will advance our understanding of microglial biology and its therapeutic potential in ischemic stroke.

## Author Contributions

**Yi‐Sha Guo:** conceptualization, writing – original draft. **Yunlin Shang:** writing – review and editing. **Jiajia Yao:** methodology, project administration. **Xia Bi:** supervision, validation. All authors have approved the final version of the manuscript.

## Consent

The authors have nothing to report.

## Conflicts of Interest

The authors declare no conflicts of interest.

## Data Availability

Data sharing is not applicable to this article as no new data were created or analyzed in this study.
